# The Role of Vitamin K and Its Related Compounds in Mendelian and Acquired Ectopic Mineralization Disorders

**DOI:** 10.3390/ijms20092142

**Published:** 2019-04-30

**Authors:** Lukas Nollet, Matthias Van Gils, Shana Verschuere, Olivier Vanakker

**Affiliations:** 1 Center for Medical Genetics, Ghent University Hospital, 9000 Ghent, Belgium; lukas.nollet@ugent.be (L.N.); matthias.vangils@ugent.be (M.V.G.); slversch.verschuere@ugent.be (S.V.); 2 Department of Biomolecular Medicine, Ghent University, 9000 Ghent, Belgium

**Keywords:** Ectopic mineralization, vitamin K, matrix gla protein, pseudoxanthoma elasticum, PXE-like syndrome, vascular calcification, chronic kidney disease, Keutel syndrome

## Abstract

Ectopic mineralization disorders comprise a broad spectrum of inherited or acquired diseases characterized by aberrant deposition of calcium crystals in multiple organs, such as the skin, eyes, kidneys, and blood vessels. Although the precise mechanisms leading to ectopic calcification are still incompletely known to date, various molecular targets leading to a disturbed balance between pro- and anti-mineralizing pathways have been identified in recent years. Vitamin K and its related compounds, mainly those post-translationally activated by vitamin K-dependent carboxylation, may play an important role in the pathogenesis of ectopic mineralization as has been demonstrated in studies on rare Mendelian diseases, but also on highly prevalent disorders, like vascular calcification. This narrative review compiles and summarizes the current knowledge regarding the role of vitamin K, its metabolism, and associated compounds in the pathophysiology of both monogenic ectopic mineralization disorders, like pseudoxanthoma elasticum or Keutel syndrome, as well as acquired multifactorial diseases, like chronic kidney disease. Clinical and molecular aspects of the various disorders are discussed according to the state-of-the-art, followed by a comprehensive literature review regarding the role of vitamin K in molecular pathophysiology and as a therapeutic target in both human and animal models of ectopic mineralization disorders.

## 1. Introduction

Ectopic mineralization disorders are characterized by abnormal mineral deposition in soft tissues, such as the skin, kidneys, blood vessels, and cardiac valves [1]. Mainly consisting of calcium hydroxyapatite, Ca_5_(PO_4_)_3_OH, these deposits greatly resemble endochondral bone both at the ultrastructural and signaling level [2]. Pathological biomineralization is caused by an imbalance between pro- and anti-mineralizing factors, which can be inherited or acquired later in life [3]. Indeed, ectopic mineralization occurs in multiple rare Mendelian disorders as well as in some of the most prevalent diseases in humans, such as diabetes mellitus, chronic kidney disease (CKD), and ischemic stroke [4]. Monogenic orphan diseases, such as pseudoxanthoma elasticum (PXE)—in which abnormal connective tissue calcification is a hallmark finding—are considered excellent research models to investigate the molecular and cellular signaling pathways involved in ectopic mineralization [4]. High quality fundamental research into these intricate genetic disorders is needed to gain further knowledge about the molecular etiopathogenesis of these diseases, identify novel therapeutic targets, and develop future drugs, which may prove to be beneficial not only to those patients carrying the burden of a rare heritable ectopic mineralization disorder, but also to the millions of people worldwide suffering from acquired, mostly cardiovascular, calcification disorders, causing significant morbidity, mortality, and associated healthcare costs both on an individual and population level. 

### 1.1. Vitamin K: Basic Chemical Structure and Function

The term ‘vitamin K’ groups several hydrophobic fat-soluble vitamins with an analogous chemical structure, i.e., methylated naphtoquinone with varying aliphatic side chains [5]. Two naturally occurring forms of vitamin K exist: Vitamin K1 (phylloquinone) is the major dietary source of vitamin K in humans and is most abundant in green leafy vegetables and vegetable oils, while vitamin K2 (menaquinones, consisting of multiple forms, such as MK-4 and MK-7) are found in highly fermented foods, like dairy products, and animal-based foods, like meat [6]. Menaquinones can also be endogenously produced by the conversion of vitamin K1 to K2 by intestinal microorganisms, such as *Bacteroides* and *Lactococcus,* albeit only accounting for a relatively small percentage of the daily vitamin K supply [6,7]. A daily vitamin K intake of 120 and 90 µg for men and women, respectively, is recommended by the United States Institute of Medicine [8]. 

**Figure 1:**
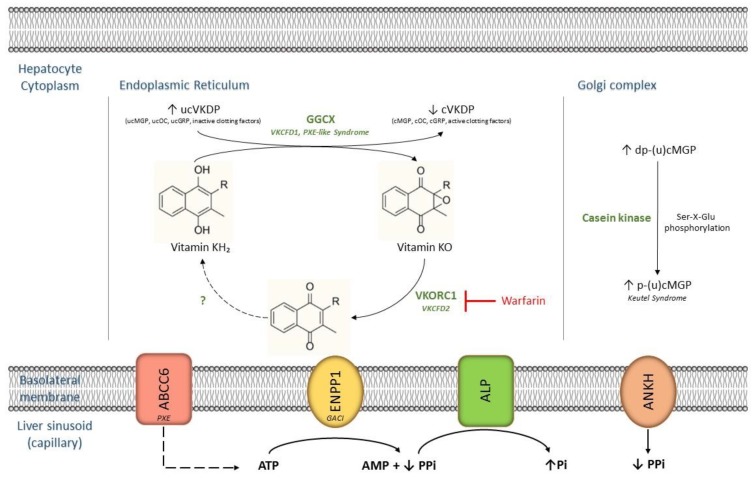
Schematic representation of vitamin K metabolism and related compounds in hepatocytes. Arrows indicate differential expression of molecular targets as observed in ectopic mineralization pathophysiology. Note that dp-ucMGP (an extrahepatic VKDP) is mainly synthesized in VSMCs and chondrocytes, and is thereafter transported to the liver. Post-translational modification then takes place in hepatocytes as shown above. ABCC6: ATP-binding cassette transporter subfamily C member 6. ALP: alkaline phosphatase. AMP: adenosine monophosphate. ANKH: progressive ankylosis homolog protein. ATP: adenosine triphosphate. ENPP1: ectonucleotide pyrophosphatase-phosphodiesterase 1. GACI: generalized arterial calcification of infancy. GGCX: gamma-glutamyl carboxylase. GRP: gla-rich protein. MGP: matrix gla protein. OC: osteocalcin. Pi: inorganic phosphate. PPi: inorganic pyrophosphate. PXE: pseudoxanthoma elasticum. VKCFD1/2: vitamin K-dependent coagulation factor deficiency 1/2. (d)(p)(u)cVKDP: (de)(phosphorylated)(un)carboxylated vitamin K-dependent protein. VKORC1: vitamin K 2,3-epoxide reductase complex subunit 1. VSMC: vascular smooth muscle cell.

After being absorbed by the digestive system and brought into the systemic circulation, vitamin K is avidly taken up by the liver, where it exerts its function as an essential co-factor for the activation of several vitamin K-dependent proteins (VKDPs) [9]. In humans and vertebrates, important VKDPs include coagulation factors II, VII, IX, and X; protein C and S; matrix gla protein (MGP); gla-rich protein (GRP); and osteocalcin (OC). All need to undergo post-translational carboxylation of glutamate (Glu) residues into γ-carboxyglutamate (Gla) to become active (Figure 1) [10]. This reaction is catalyzed by the endoplasmic enzyme gamma-glutamyl carboxylase (GGCX) and requires a reduced hydroquinone form of vitamin K, hence the term ‘vitamin K-dependent’ [6,10]. As a result of the carboxylation reaction, reduced vitamin K is transformed into an epoxide, which then needs to be recycled back to the reduced form [6]. This process is catalyzed by the vitamin K 2,3-epoxide reductase complex subunit 1 (VKORC1)—the main target of the frequently used anticoagulant drug, warfarin—followed by various reduction pathways, which are still not very well understood to date [6]. 

Loss-of-function mutations in the genes encoding the enzymes involved in this so-called vitamin K cycle, such as *GGCX* and *VKORC1*, result in rare heritable disorders with extensive phenotypic variability, e.g., the PXE-like syndrome with multiple coagulation factor deficiency, and isolated vitamin K-dependent coagulation factor deficiencies (VKCFD1 and VKCFD2) [11]. Recently, it was found that missense mutations in *VKORC1* cause extensive medial arterial calcification in rats, linking this enzyme not only to the coagulation cascade, but also to the regulation of biomineralization [12]. 

### 1.2. Vitamin K is Associated with Ectopic Mineralization

**Figure 2:**
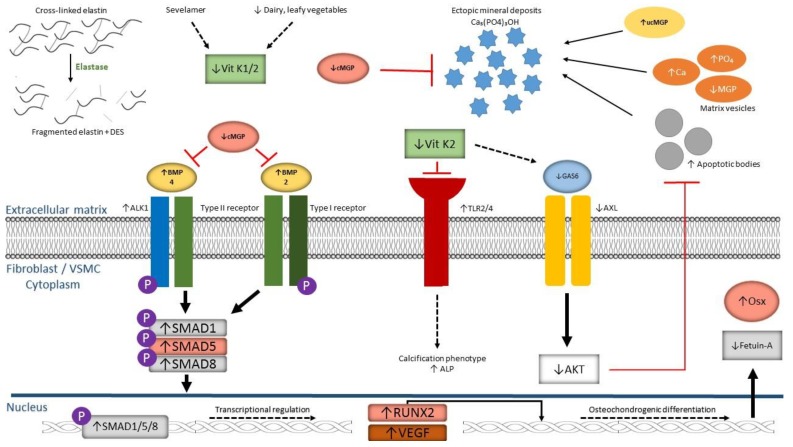
Schematic representation of molecular alterations in the extracellular matrix and cytoplasm of fibroblasts/vascular smooth muscle cells contributing to ectopic mineralization, focusing on vitamin K and related compounds. AKT: AK strain transforming. ALK1: activin receptor-like kinase 1. ALP: alkaline phosphatase. AXL: AXL receptor tyrosine kinase. BMP: bone morphogenetic protein. Ca: calcium. Ca_5_(PO_4_)_3_OH: calcium hydroxyapatite. DES: (iso)desmosine. GAS6: growth arrest specific-6. (u)cMGP: (un)carboxylated matrix gla protein. OSX: osterix. PO_4_: phosphate. RUNX2: runt-related transcription factor 2. SMAD: small body size mothers against decapentaplegic. TLR: toll-like receptor. VEGF: vascular endothelial growth factor. Vit K1/2: vitamin K1/2.

A potential role for vitamin K and its related compounds in the pathogenesis of ectopic mineralization disorders was suggested by two distinct findings: I) *GGCX* mutations cause a rare calcification phenotype similar to that of PXE, but accompanied by deficiencies in vitamin K-dependent clotting factors; and II) PXE patients have significantly lower serum levels of vitamin K compared to the reference population (Figure 2) [10,13]. 

MGP, a strong vitamin K-dependent mineralization inhibitor, has been attributed a crucial role in these pathological calcification processes [14]. *Mgp* knockout mice die within 8 weeks of birth due to extensive vascular mineralization and subsequent blood vessel rupture [15]. In humans, homozygous mutations in the *MGP* gene cause Keutel syndrome, which is characterized by abnormal cartilage calcification, peripheral pulmonary stenosis, and midfacial hypoplasia [16]. Intriguingly, a clinically indistinguishable syndrome can be observed in newborns suffering from warfarin-induced embryopathy, caused by maternal use of the coumarin derivative warfarin between gestational week 6 and 9 [11]. Dysfunctional γ-glutamyl carboxylation of MGP due to warfarin-mediated inhibition of the vitamin K cycle has thus been proposed as the main cause of this disorder [11]. In vascular mineralization, specific antibodies targeting uncarboxylated MGP (ucMGP) have demonstrated colocalization of ucMGP with calcification deposits in the blood vessel wall, hence indicating a potential role for ucMGP and carboxylated MGP (cMGP) as biomarkers of vascular mineralization in patients [17].

MGP was found to be primarily synthesized by vascular smooth muscle cells (VSMCs), chondrocytes, endothelial cells (ECs), and fibroblasts [18]. Following γ-glutamyl carboxylation, which confers a high affinity for mineral ions, such as calcium, phosphate, and hydroxyapatite, in the endoplasmic reticulum (ER), additional serine phosphorylation of repeated Ser-X-Glu sequences at the N-terminus end—a process thought to be catalyzed by casein kinase in the Golgi apparatus—is needed for MGP to obtain its full anti-mineralizing capacity (coined p-cMGP) [19].

A comprehensive overview of the specific molecular mechanisms by which MGP is able to prevent ectopic calcification can be found in the review article of Schurgers et al. [20]. They include the regulation of matrix vesicles and apoptotic bodies (ABs) originating from VSMCs, inhibition of hydroxyapatite formation by direct binding of MGP to the crystal deposits via its negatively charged Gla residues and phosphorylated serines, and inhibition of VSMC transdifferentiation to an osteochondrogenic phenotype [20]. 

Indeed, MGP may possess anti-apoptotic properties that prevent the formation of Abs, which forms an initial nidus for calcification and crystal growth, from VSMCs [21,22]. This protective effect is thought to be caused by the binding of fully carboxylated MGP to bone morphogenetic protein-2 (BMP-2), thereby limiting its pro-apoptotic and osteo-inductive function [23]. 

In this review article, a comprehensive summary will be given on both heritable and acquired ectopic mineralization disorders characterized by dysfunctional vitamin K homeostasis. Clinical and molecular aspects are described as well as vitamin K-associated therapeutic interventions in both human and animal models, according to the current state-of-the-art. 

## 2. Mendelian Ectopic Mineralization Diseases and Vitamin K 

### 2.1. Pseudoxanthoma Elasticum

#### 2.1.1. Phenotype

Pseudoxanthoma elasticum (PXE; OMIM #264800) is an autosomal recessive ectopic mineralization disorder characterized by calcification and fragmentation of elastic fibers in the skin, eyes, and cardiovascular system [24,25]. Previously known as the Grönblad–Strandberg syndrome, PXE causes significant morbidity and occasional mortality, mainly due to cardiovascular complications [26].

While the exact prevalence of PXE is unknown, it is estimated that 1 in 25,000 to 1 in 100,000 individuals are affected, with a frequency of heterozygote carriers in the normal population ranging between 1:66 and 1:160 [24]. Female individuals are two times more likely to develop PXE than their male counterparts, though the reasons for this remain unknown [26]. 

Clinical manifestations are usually detected from the second or third decade onwards, while being rarely present at birth [25,26]. The first to occur are usually the cutaneous manifestations of PXE, consisting of small xanthoma-like yellowish papules or larger plaques formed by coalesced papules [27,28]. These lesions typically appear first on the lateral side of the neck while other regions, mainly flexural zones, such as the axillae, antecubital, and popliteal fossae, groins, and peri-umbilical skin, become affected during further disease progression [28]. 

If a skin biopsy is taken, histopathological examination using conventional hematoxylin-eosin (HE) staining reveals distinct microscopic features, such as fragmentation and clumping of short and thick elastic fibers, typically located in the mid-dermis. Alternative staining procedures, like Von Kossa, Verhoeff–Van Gieson, and Alizarin Red, show pronounced calcium deposits and an increased amount of elastin in the dermis [27]. Furthermore, abnormal collagen fibrils can be observed near the calcified elastic fibers as well as abnormal amounts of proteoglycans and glycosaminoglycans [29]. Numerous fibroblasts and macrophages surround the calcified deposits [26]. Interestingly, biopsies taken from a macroscopic normal skin site may show the same histopathological changes as a biopsy taken from a macroscopic lesion though this is not always the case [28]. 

The earliest ocular manifestation of PXE which can be seen with funduscopy is peau d’orange, a lesion consisting of small dark spots mainly localized temporal to the macula [24,26]. Later in life, elastic fibers in Bruch’s membrane of the eye become progressively calcified and fragmented, causing cracks in the membrane that manifest as angioid streaks, dark lines extending outward from the optic disk [27]. Due to this fragmentation of Bruch’s membrane, choroidal neovascularization (CNV), in which newly formed, but fragile blood vessels penetrate the membrane, is able to take place. This process has been found to be primarily mediated by vascular endothelial growth factor (VEGF) [24]. Fluid leakage or hemorrhage of these vessels may cause metamorphopsia and may further decrease vision, while afterwards, the formation of macular scars may lead to a loss of central vision and even blindness [25].

Cardiovascular manifestations usually occur after the onset of cutaneous and ocular manifestations, typically from the fifth decade of life onwards [30]. They are caused by dystrophic calcification of the tunica intima and media of mid-sized arteries, resulting in occlusive vessel disease [27].

PXE patients may therefore suffer from angina pectoris, reduced pulse amplitude, arterial and renovascular hypertension, intermittent claudication, gastro-intestinal hemorrhage, restrictive cardiomyopathy, valvular heart disease, and sudden cardiac death [24,26]. Early-onset arteriosclerosis due to accelerated vascular calcification (VC) may result in early myocardial infarctions and ischemic cerebrovascular disease, of which the risk is almost 4 times greater in PXE patients than in healthy controls [28,31]. 

Remarkably, heterozygous carriers are also at increased risk of developing cardiovascular disease, such as coronary artery disease (CAD), peripheral arterial disease (PAD), and ischemic stroke, when compared to the general population [25,30,32,33].

To date, medical management of PXE is mostly symptomatic (intraocular anti-VEGF treatment; primary and secondary cardiovascular prevention) as no curative treatment options are available yet, warranting further research into the pathogenic mechanisms of this disorder.

#### 2.1.2. Molecular Etiology

Central in the etiopathogenesis of PXE are mutations in the *ABCC6* gene (ATP-binding cassette transporter, subfamily C member 6), formerly known as *MRP6* (multi-drug resistance-associated protein 6), encoding a transmembrane transporter of which the substrate is currently unknown [24]. ABCC6 is primarily expressed in the liver and kidneys while, remarkably, expression is low in tissues affected by PXE, such as the skin, vessel walls, and Bruch’s membrane of the eye, adding to the hypothesis that PXE is a metabolic disorder caused by a decrease in plasma and tissue concentrations of an ABCC6-transported or -related factor [24].

The ABCC6 efflux transporter has been shown to be involved in the release of intracellular nucleotides, such as ATP, over the sinusoidal membrane of hepatocytes [1,34,35]. Secreted ATP is then converted into AMP and PPi (inorganic pyrophosphate), yet still within the liver vasculature as shown by in vivo mice liver perfusion experiments [34]. This reaction is catalyzed by the ectonucleotide pyrophosphatase-phosphodiesterase 1 (ENPP1). Loss-of-function mutations in the *ENPP1* gene cause generalized arterial calcification of infancy (GACI; OMIM #208000), a very severe vascular calcification disorder leading to premature death in the affected newborn [3,34,36,37].

As PPi has been proven to be a pivotal calcification inhibitor, reduced PPi concentrations greatly contribute to the pathological mineralization found in PXE and other connective tissue calcifying disorders (CTCs) [38]. Indeed, plasma PPi concentrations in PXE patients were found to be 2.5-fold lower than in healthy controls, though significant variability seems to exist [34]. Similar results were found in PXE fibroblast cultures and in the plasma of *Abcc6*^−/−^ mice [35,38].

Increased expression of alkaline phosphatase (ALP; *TNAP*), which breaks down PPi to inorganic phosphate (Pi), may also contribute to decreased PPi levels. In PXE, a more than 3-fold increase in ALP expression compared to healthy controls was found by Hosen et al. [39].

However, though an altered Pi/PPi ratio may well be involved in the process of ectopic mineralization, it does not completely explain the specific multisystemic PXE phenotype as altered Pi/PPi ratios are also present in other CTCs, such as GACI (a prenatal acute vascular phenotype) and arterial calcification due to CD73 deficiency (a chronic vascular and joint phenotype) [40]. Furthermore, a study by Zhao et al. [41] showed that although *Enpp1* overexpression in *Abcc6*^−/−^ mice was able to increase plasma PPi levels and significantly reduce mineralization, small mineralization foci were still detectable in these mice, while overexpression of *Enpp1* in mutant *Enpp1^asj^* mice completely normalized plasma PPi levels and fully prevented ectopic mineralization. They therefore suggested that a yet unknown PPi-independent ABCC6 mechanism is also at play for the prevention of ectopic mineralization [41]. 

Recently published data by Van Gils et al. [42] showed that a deficiency of the transmembranary PPi transporter progressive ankylosis homolog protein (ANKH) further contributes to the already decreased plasma PPi levels in PXE patients. Also, in this study, it was found that multiple cellular signaling cascades are affected in PXE, mainly those revolving around transforming growth factor-βs (TGF-βs) and bone morphogenetic proteins (BMPs) [39,42]. 

#### 2.1.3. Vitamin K: Role and Therapeutic Options in PXE

The role of vitamin K in the etiopathogenesis of PXE has been a topic of some controversy in the literature. In 2010, a study by Vanakker et al. [10] showed that PXE patients have decreased vitamin K serum levels compared to the reference population. Furthermore, a significant reduction in serum and plasma total ucMGP concentration was found in PXE patients compared to healthy controls [10]. Immuno-transmission electron microscopy of PXE skin biopsies using anti-ucMGP and -cMGP antibodies revealed a distinct appearance with anti-ucMGP antibodies mainly found in the core of the elastic fibers, whereas the scarce anti-cMGP antibodies were precisely localized at the border of the mineralized areas, clearly separating this pathological region from normal elastin fibers [10]. Vanakker et al. thus concluded that cMGP is an important inhibitor of ectopic mineralization and that decreased activity of this carboxylated protein in PXE is mainly caused by a deficiency of the carboxylation co-factor vitamin K [10].

In the *Abcc6*^−/−^ murine model, similar results had been obtained by Li et al. [43] showing reduced MGP serum concentrations in knockout mice compared to wild type (WT) mice. MGP isolated from the liver of *Abcc6*^−/−^ mice was mostly undercarboxylated, thus proving dysfunctional vitamin-K dependent carboxylation in the organ with normally the highest expression of the Abcc6 transporter in WT mice [43].

Immunohistochemical (IHC) staining of *Abcc6*^−/−^ muzzle skin containing the vibrissae with its characteristic pathological mineral deposits showed colocalization of anti-ucMGP antibodies with the characteristic pathological calcified areas, while staining with anti-cMGP antibodies was completely negative [43].

Regarding the activity levels and concentrations of other VKDPs, like OC, which may be negatively influenced by vitamin K deficiency in PXE patients, Vanakker et al. [10] did not detect abnormal OC levels in PXE serum samples, while interestingly, OC levels seemed to be highly disturbed in IHC stained skin lesion biopsies from PXE patients compared to controls [10]. This peculiar finding may be explained by the high expression of low-density lipoprotein receptor-related protein 1 (LRP1) by osteoblasts, which allows for highly efficient uptake of vitamin K-rich chylomicron remnants containing apolipoprotein E, thus resulting in an increased biosynthesis of OC in localized areas of calcification without changing OC levels in the systemic circulation [44].

As mentioned earlier, full carboxylation of MGP is needed to ensure adequate binding and inhibition of BMP-2, an important protein involved in osteochondrogenic transdifferentiation of fibroblasts and VSMCs [23]. Interestingly, Hosen et al. [39] showed a significant upregulation of the BMP-2–SMAD1/5/8–RUNX2 pathway in PXE fibroblasts and affected human and murine dermal tissues compared to controls, using mRNA expression profiling and IHC staining. Additional immune co-staining of *Abcc6*^−/−^ muzzle skin showed co-localization of the labelled antibodies with mineralization foci visualized by Alizarin Red staining [39]. It may therefore be assumed that decreased vitamin K-dependent carboxylation of MGP in PXE patients results in decreased inhibition of BMP-2, which then, through its main SMAD-mediated signaling pathway, causes a shift towards an osteogenic phenotype in fibroblasts and VSMCs (Figure 2). 

Another bone morphogenetic protein, i.e., BMP-4, has been linked to MGP and may be of interest to PXE pathogenesis. As described in the review of Schurgers et al. [20], BMP-4 has been found to induce activin receptor-like kinase 1 (ALK1), resulting in increased VEGF synthesis and secretion [20]. By binding BMP-4, cMGP is able to suppress the ALK1-mediated secretion of VEGF [20]. Conversely, decreased levels of cMGP as found in PXE may result in increased expression of VEGF, which is suggested to play a major role in the pathogenesis of CNV and subsequent vision loss in PXE patients as repeated intravitreal injections of anti-VEGF antibodies, like bevacizumab, were found to be very effective in treating this specific ocular complication [45,46,47]. In conclusion, the MGP–BMP-2 or MGP–BMP-4 axis may potentially be an interesting target for novel therapeutic agents although further research is currently needed. 

In a study from 2013, Li et al. [48] further established the role of vitamin K in PXE by feeding *Abcc6* ^−/−^ mice a diet enriched with high concentrations of the anticoagulant drug, warfarin, the most prescribed vitamin K antagonist used in clinical practice. A known side-effect of chronic warfarin treatment in patients suffering from atrial fibrillation is increased ectopic calcification of cardiac valves, coronary arteries, and peripheral blood vessels [49,50,51]. *Abcc6* deficient mice placed on the warfarin-supplemented diet exhibited a significantly (16-fold) increased degree of ectopic mineralization in multiple organ systems, like kidneys, heart, aorta, and eyes, compared to WT mice fed the warfarin-rich diet and *Abcc6*^−/−^ mice fed the control diet [48]. A massive accumulation of mineral deposits in the treatment group was accompanied by a significant increase in ucMGP observed on IHC-stained aorta tissue sections, as could be expected from the vitamin K-antagonizing properties of warfarin [48]. An additional survey of 539 PXE patients revealed that 2.6% of patients had previous or current use of warfarin, potentially exposing them to a high risk of accelerated ectopic calcification [48]. The results obtained in this study may therefore have important clinical implications as the protective effect of warfarin regarding ischemic stroke, which has a highly increased incidence in PXE patients, should be weighed against the potentially harmful side effect of rapidly increasing ectopic mineralization. 

As vitamin K deficiency and/or dysfunctional metabolism thus appeared to play an important role in PXE, various vitamin K substitution experiments have been conducted throughout the years, trying to halt, prevent, or even reverse the formation of mineral deposits in PXE patients and animal models. Brampton et al. [52] compared the amount of ectopic calcification in *Abcc6*^−/−^ mice placed on a standard rodent diet supplemented with either vitamin K1 or MK-4 (vitamin K2) to *Abcc6*^−/−^ mice fed only the standard diet. Three different *Abcc6*^−/−^ groups were created, i.e., in utero, pre-onset of mineralization, and post-onset of mineralization exposure to the vitamin K1- or MK4-enriched diet. Although increased vitamin K1 and K2 serum levels were observed in the treatment group, vitamin K supplementation failed to counteract the mineralization process as no difference was found between the amount of calcification in the treatment animals compared to the control animals [52]. Interestingly, *Abcc6*^−/−^ mice receiving a high dose MK-4 enriched diet had marked liver discoloration with small lesions on the hepatic surface, while no such side-effects were found in *Abcc6*^−/−^ mice fed with high doses of vitamin K1 [52]. Abcc6 deficiency may thus be associated with a dysfunctional metabolism of vitamin K2, but not K1, in the PXE mouse model. 

Similar results were obtained by Gorgels et al. [9] and Jiang et al. [53] as neither supplementation of MK-7 (vitamin K2) nor intermediate vitamin K3 conjugate in *Abcc6*^−/−^ mice could halt the pathologic mineralization process.

Regarding other animal models, the *abcc6a*-mutant gräte zebrafish (*Danio rerio*) was used in a vitamin K1 supplementation experiment of Mackay et al. [54] with remarkable results. Indeed, supplementation of vitamin K1 to the media of 4 to 8 days post-fertilization mutant embryos resulted in a marked reduction of the axial hypermineralization normally observed in gräte zebrafish, with a significant rescue of the phenotype [54]. Similar results were obtained in *enpp1*^−/−^ zebrafish, while the addition of warfarin to the medium resulted in a 2-fold increase in mineralization of both genotypes [49]. The ability of vitamin K1 to counteract ectopic mineralization in a PXE zebrafish model, but not in a PXE mouse model definitely warrants further research. 

Finally, Carrillo-Linares et al. [55] very recently studied the effect of parenteral administration of a single dose of vitamin K1 in PXE patients versus controls, measuring baseline and 1 and 6 weeks post injection vitamin K levels, urinary concentration of vitamin K metabolites, and serum concentration of carboxylated OC. Higher serum MK-4 levels at baseline were detected in PXE patients compared to controls, with a significant increase in MK-4 concentration at 1 and 6 weeks post-vitamin K injection, indicating that the conversion of vitamin K1 to vitamin K2 (MK-4) is conserved in PXE patients [55]. Urinary vitamin K metabolites were found to be lower in PXE patients at baseline compared to controls, a difference which was maintained after vitamin K administration [55]. Increased serum levels of carboxylated OC after vitamin K administration in PXE patients was also noted, suggesting preserved vitamin K-dependent γ-glutamyl carboxylation. Similar serum levels of PIVKA-II (protein induced by vitamin K absence or antagonist-II, uncarboxylated prothrombin) were measured in PXE patients compared to controls, confirming that the vitamin K-dependent coagulation factors are not affected in PXE, contrary to PXE-like syndrome (see below) [55]. 

### 2.2. PXE-Like Syndrome with Multiple Coagulation Factor Deficiency

#### 2.2.1. Phenotype

This disorder (OMIM # 610842), first comprehensively described by Vanakker et al. [13] in 2007, greatly resembles PXE as it is clinically characterized by yellow skin papules, angioid streaks in the eye, and occasionally symptoms of peripheral artery disease, such as intermittent claudication. However, it was noted that these patients developed more excessive skin folding than is usually observed in PXE, spreading further along the abdomen and limbs [13,56,57]. Ophthalmologic examination revealed intact visual acuity in all patients, indicating a milder retinopathy than in PXE [13,56]. 

Most interestingly, a prolonged prothrombin time with a significant deficiency of vitamin K-dependent coagulation factors was found in the serum of these patients [13,56,57]. Clinical manifestations included meningeal, vaginal and postpartum hemorrhages as well as spontaneous hematemesis and gingival bleeding, although the majority of patients had not experienced any abnormal bleeding events [13]. 

#### 2.2.2. Molecular Etiology

Histopathological analysis of skin biopsies using light microscopy could not differentiate this novel clinical phenotype from PXE, whereas electron microscopic imaging revealed calcified deposits only at the periphery of affected elastic fibers while in PXE these deposits always occur in the core of elastic fibers [13]. Finally, genetic analysis found bi-allelic mutations in the *GGCX* gene to be responsible for the disorder, while no *ABCC6* mutations were identified [13]. It should be noted, however, that an important clinico-genetic overlap may exist, as was demonstrated by a case report of Vanakker et al. [58] describing a patient in which signs and symptoms of both PXE and PXE-like syndrome were observed together with two *ABCC6* mutations and a gain-of-function SNP in the *GGCX* gene, supporting the hypothesis that PXE-like syndrome is part of a wider spectrum of ectopic calcification disorders related to PXE. 

As mentioned earlier, loss-of-function mutations in *GGCX* can also result in the very rare bleeding disorder, VKCFD1 [11]. The genetic overlap between VKCFD1 and PXE-like syndrome, though a distinct phenotypic presentation, may be explained by the fact that mutations causing PXE-like syndrome seem to typically occur in exons 8, 10, and 12 of the *GGCX* gene, while no such mutations have been found so far in VKCFD1 [13]. It may therefore be hypothesized that these specific exons code for GGCX domains involved in the activation of both coagulation factors and other Gla proteins, such as MGP or OC, whereas VKCFD1-causing mutations only interfere with the activation of vitamin K-dependent coagulation factors [13]. This hypothesis was endorsed by the systematic review of De Vilder et al. [59] demonstrating genotype–phenotype correlations between mutations in distinct GGCX functional domains and clinical manifestations, such as cardiac involvement. 

#### 2.2.3. Role of Vitamin K in the PXE-Like Syndrome

IHC staining of a lesional skin biopsy from patients suffering from PXE-like syndrome revealed a marked presence of ucMGP colocalizing with mineral deposits in the mid-dermal elastorrhexis area, while cMGP expression was almost absent [56,60]. Furthermore, significantly increased ucMGP serum levels were found in PXE-like patients accompanied by a very high ucMGP/cMGP ratio, while no such differences could be observed in classic PXE patients compared to controls [10]. These interesting findings may suggest an even greater role for dysfunctional vitamin K metabolism in the pathogenesis of PXE-like syndrome compared to PXE, which can be easily assumed as the decreased VKDP activity in PXE-like syndrome being caused by the direct effect of an abnormally functioning GGCX enzyme due to loss-of-function mutations. On the contrary, serum vitamin K levels were found to be unaltered in PXE-like patients compared to healthy controls, indicating a sufficient supply of this cofactor in PXE-like syndrome [10]. 

Given the limited amount of reported PXE-like syndrome cases, novel research results and insights into this disorder are scarce, particularly when concerning molecular signaling pathways. Further basic research is thus of uttermost importance in order to elucidate the role of GGCX dysfunction and GGCX-related vitamin K abnormalities in ectopic calcification disorders. 

### 2.3. Keutel Syndrome

#### 2.3.1. Phenotype

Keutel syndrome (KS; OMIM # 245150) is an extremely rare autosomal recessive disorder caused by loss-of-function mutations in the *MGP* gene [16]. First described by Keutel et al. [61] in 1971, approximately 30 KS patients have been identified so far with a mean age of 8 years at the time of diagnosis [3,62]. Clinically, KS is characterized by abnormal calcification of cartilage (e.g., cricoid and rib cartilage), peripheral pulmonary artery stenosis, brachytelephalangy (short distal phalanges), hearing loss, and dysmorphic facial features, such as midface hypoplasia or depressed nasal bridge [61,62,63,64,65]. Interestingly, while *Mgp* knockout mice die within 2 months due to massive arterial calcification and aortic rupture, clinical and histological signs of VC have only been demonstrated in one KS patient so far, i.e., the original patient described by Keutel et al. [61]. HE and Von Kossa staining of tissue samples obtained during postmortem examination of this patient revealed concentric VC in medium-sized branches of the pulmonary arteries as well as in coronary and skin arteries [62]. Additionally, fragmented and calcified mid-dermal elastic fibers were found in skin biopsies from multiple KS patients, a histopathological image reminiscent of other ectopic mineralization disorders, in particular PXE [62,66].

The life expectancy of KS patients mainly depends on the severity of associated respiratory complications, which are often refractory to standard therapies, such as corticosteroids or bronchodilators [65,67]. 

#### 2.3.2. Molecular Etiology and Role of Vitamin K

To date, only a handful of different mutations in the *MGP* gene causing the KS phenotype have been identified, mostly resulting in splice site anomalies and/or premature termination of *MGP* translation [64]. Regarding MGP activity, Cranenburg et al. [62] demonstrated increased serum levels of phosphorylated MGP and decreased levels of carboxylated MGP in a KS patient compared to his parents (heterozygous carriers) and unaffected sibling (wild type). Additional daily vitamin K1 supplementation for 3 months did not significantly alter ucMGP or cMGP serum levels in the proband, despite a marked decrease in the ucOC/cOC ratio indicating an improvement in the patient’s systemic vitamin K status [62]. 

The authors hypothesized that the high levels of pMGP in the proband might be the result of a relatively increased biosynthesis of MGP due to the high calcification load [62]. Additionally, dysfunctional MGP carboxylation in KS might be caused by the detrimental effects of the pathogenic mutations themselves, as they often result in heavily distorted protein structures, e.g., in the KS patient of Cranenburg et al. [62], only the first 14 out of 84 MGP amino acids were comparable to the wild type protein. As phosphorylated MGP might be able to bind calcium crystals in the relative absence of cMGP, the authors further hypothesized that increased pMGP levels found in KS patients may prevent the development of a severe vascular mineralization phenotype [62]. Finally, the results obtained by Cranenburg et al. [62] indicate that vitamin K supplementation might not be beneficial in the treatment of KS patients, although further research in a larger patient cohort is needed. 

## 3. Acquired (Vascular) Calcification Disorders and Vitamin K

### Phenotypes and Vitamin K Supplementation

VC has long been considered a passive and clinically irrelevant process resulting in the accumulation of mineral deposits in existent atherosclerotic intima plaques and/or the medial vessel wall [68,69]. However, increasing insights into the pathogenesis of VC have demonstrated that this specific type of ectopic mineralization is in fact an actively controlled multifactorial process and a major cause of cardiovascular disease worldwide [70].

Molecular alterations observed in VC pathogenesis include: I) Differentiation of VSMCs to an osteoblast-like phenotype; II) release of calcifying matrix vesicles rich in calcium and phosphate, but poor in mineralization inhibitors, like MGP; III) increased expression of osteochondrogenic markers, like BMP-2, Osterix (Osx), and RUNX2; and IV) decreased expression of calcification regulators, such as fetuin-A and ANKH (see above, PXE) and V), increased ER stress, and apoptosis [69,70,71]. 

The role of vitamin K and VKDPs in VC has been relatively well established, specifically regarding medial VC. Medial calcification, also known as Monckeberg’s arteriosclerosis, is a common finding in elderly patients as a result of physiological aging, but is also present in patients suffering from diabetes, CKD, or heritable mineralization disorders, like PXE [32,69,72,73]. Advanced medial calcification may give rise to systolic hypertension, myocardial infarction, peripheral artery disease, and calciphylaxis, a highly fatal necrotizing skin condition due to inadequate blood supply mostly observed in hemodialysis patients [69,74]. Vitamin K-deficiency is commonly found in these at-risk populations, and low vitamin K status has been proven to be associated with calcification and cardiovascular disease, e.g., peripheral artery disease [75]. Dialysis patients in particular seem to be vulnerable to subclinical vitamin K-deficiency as they are commonly advised to consume a diet low in potassium (leafy green vegetables, rich in vitamin K1) and low in phosphate (dairy products, rich in vitamin K2), while being prescribed drugs that lower the intestinal uptake of vitamin K, such as the oral phosphate binder, sevelamer [76]. 

Most importantly, as calcification-associated cardiovascular events are still the major cause of death in the dialysis population, the clinical relevance of vitamin K deficiency in these patients might be much greater than was previously assumed [6]. Indeed, an observational cohort study by Delanaye et al. [77] found that dp-ucMGP serum levels were significantly correlated with a vascular calcification score in patients receiving hemodialysis, further supporting the role of increased ucMGP expression due to vitamin K-deficiency in the development of vascular mineralization.

A recently published study by Silaghi et al. [78] further established the role of increased MGP expression in VC pathophysiology by demonstrating significantly higher serum total MGP concentrations in patients with vascular diseases compared to healthy controls (106 vs. 51 µg/L, *p* < 0.001). This finding definitely warrants further research as the serum total MGP may thus serve as a biomarker that can reliably discriminate between healthy individuals and VC-affected patients, independent of associated traditional cardiovascular risk factors, such as smoking [78]. Additionally, the novel T50-test, which determines the transformation time point, T50, from amorphous to crystalline calciprotein particles (transporting calcium, phosphate and calcification-inhibiting proteins in serum) in artificially supersaturated serum, has proven to be closely associated with aortic stiffening and cardiovascular mortality and may thus potentially be used in the future to guide clinical decision making and therapeutic strategies in patients with a high systemic calcification propensity [79]. 

Although the exact molecular mechanisms by which vitamin K is able to reduce VC are still largely unknown to date, interesting experimental results have been published in recent years. Qiu et al. [80] showed that enriching the calcification-inducing medium of primary cultured VSMCs with vitamin K2 resulted in a significantly reduced mineralization content compared to control VSMC cultures, mainly by restoring growth arrest specific-6 (Gas6) expression and its downstream Axl/Akt anti-apoptotic signaling pathway. In a study by Wang et al. [81], vitamin K2 was found to suppress the expression of Toll-like receptors 2 and 4 (TLR2–TLR4) in *Apoe^−/−^* mice, resulting in a significant reduction of atherosclerotic plaque formation and aortic intima calcification compared to *Apoe^−/−^* mice in the control group (who developed the typical vascular phenotype of extensive atherosclerosis with associated intima calcification). Furthermore, decreased expression of ALP, which metabolizes PPi, was found in aortic tissue sections from the treatment group [81]. As mentioned earlier, reduced PPi concentrations caused by increased ALP activity is a well-established pathogenic factor in several Mendelian ectopic mineralization disorders, like PXE and GACI, and may thus form a common causal and potential pharmaceutically targetable pathway linking heritable to acquired calcification disorders. 

In the last decade, a novel VKDP which may play an important role in VC has been discovered, namely Gla-rich protein (GRP), which has the highest density of Gla-residues of all known VKDPs, resulting in a high affinity for calcium [68]. Bordoloi et al. [70] comprehensively reviewed the role of GRP in a variety of pathophysiological conditions, like ectopic mineralization, osteoarthritis, and breast carcinoma. In summary, increased uncarboxylated GRP (ucGRP) expression has been found at sites of osteogenic deposits, like in calcified aortic valves or PXE skin lesions, while carboxylated GRP (cGRP) was shown to inhibit VC to the same extent as cMGP [68,82]. Upregulation of α-smooth muscle actin and downregulation of osteopontin (OPN) seemed to be important pathways by which cGRP is able to inhibit VC [82]. More recently, cGRP has been found to counteract VC by inhibiting the BMP2-SMAD1/5/8 osteochondrogenic signaling pathway in VSMCs, while a VSMC-VC phenotype induced by serum calciprotein particles isolated from CKD stage 5 patients could be rescued by incubation of the particles with cGRP [83,84]. Further research regarding the role of GRP in VC pathophysiology and its potential use as a therapeutic agent is needed.

Finally, continuing on the increasing knowledge regarding the role of MGP and vitamin K in the pathogenesis of VC, Janssen et al. [85] postulated an intriguing hypothesis coined the ‘Vitamin K deficit and elastolysis theory’. Calcification of elastic fibers, as observed in pathologic conditions, like VC and PXE, and which can be inhibited by MGP, leads to subsequent degradation of these fibers by specific proteases called elastases. Upon degradation of cross-linked elastin, the unique amino acids, desmosine and isodesmosine (DES), are released into the systemic circulation and their plasma levels could therefore be used as a universal indicator of the rate of elastin degradation in the human body. In earlier research, a positive correlation had been found between nonphosphorylated ucMGP (dp-ucMGP) serum levels and serum DES levels in patients with chronic obstructive pulmonary disease (COPD) compared to healthy controls [85]. These results thus indicate that vitamin K deficiency, resulting in high dp-ucMGP levels, is associated with increased elastin degradation. As vitamin K supplementation causes a reduction in dp-ucMGP concentrations (see below), Janssen et al. [85] hypothesized that supplementing vitamin K to the normal diet of affected individuals might also reduce the rate of elastin degradation and therefore halt disease progression in patients suffering from elasto-degenerative diseases, like COPD and cystic fibrosis. Their research group is currently evaluating their hypothesis by determining plasma dp-ucMGP and DES levels in patients with alpha-1 antitrypsin deficiency (AATD), a pulmonary disease characterized by highly accelerated elastin degradation in the lungs. In following steps, the effect of vitamin K supplementation on plasma DES levels will be evaluated and eventually compared to the current standard therapy, i.e., alpha-1 antitrypsin supplementation, which is a very costly treatment and requires weekly intravenous admissions [85]. Results from this study are highly anticipated although further research is certainly needed to evaluate whether these proposed mechanisms also apply to the pathogenesis of non-pulmonary ectopic mineralization disorders, like PXE and VC. 

Regarding the potential therapeutic use of vitamin K supplementation in patients with acquired VC disorders, multiple clinical trials are currently ongoing that are evaluating the clinical efficacy and survival benefit of vitamin K supplementation in CKD and dialysis patients, as well as in other patient groups, like type 2 diabetics and patients exhibiting marked coronary artery calcification [5,86,87]. A recently published large scale systematic review and meta-analysis by Lees et al. [88] found that vitamin K supplementation was associated with a significant reduction in calcification and dp-ucMGP concentrations compared to controls, while longitudinal studies could even demonstrate an association between VKDP serum levels and cardiovascular disease and mortality. However, due to the substantial heterogeneity of studies included in the meta-analysis, the authors still advised that larger clinical trials be conducted to evaluate the effect of vitamin K supplementation on primary endpoints, like (cardiovascular) mortality, before routine supplementation of vitamin K can be advised in daily clinical practice to high risk patients [88]. 

## 4. Conclusions

In recent years, important insights have been gained regarding the pathophysiology of ectopic mineralization by studying both rare Mendelian disorders as well as some of the most prevalent diseases worldwide, such as CKD or VC. Due to their significant morbidity and mortality, fundamental research into the underlying pathologic and molecular mechanisms is of uttermost importance as current treatment options are limited. 

In this review, we highlighted the importance of deficient γ-carboxylation of several vitamin K-dependent proteins, in particular the potent calcification inhibitor, MGP, which has greatly contributed to a better understanding of the precise molecular mechanisms leading to the abnormal deposition of calcium crystals in connective tissues. 

Although promising, in vitro and in vivo experiments that have attempted to restore vitamin K status by supplementing this apparently crucial vitamin have thus far failed to deliver convincing evidence on a potential beneficial effect of vitamin K substitution on the ectopic mineralization process in animal models or affected patients. However, further basic and clinical research into this subject is warranted, as only limited data exist today due to the rarity of these orphan diseases. Furthermore, the possible preventive effect of vitamin K on elastolysis has almost not been touched on in Mendelian calcification diseases, in which the elastic fiber is often the prime target of mineral precipitation. Results from multiple large clinical trials evaluating the effect of vitamin K substitution on ectopic mineralization in patients suffering from highly prevalent diseases, like CAD or CKD, are to be expected in the near future and may potentially boost further research into the intricate role of this macromolecule in both physiologic and pathologic processes inside the human body. 

## Abbreviations

AATD	alpha-1 antitrypsin deficiency
AB	apoptotic body
ABCC6	ATP-binding cassette transporter subfamily C member 6
AKT	AK strain transforming
ALK1	activin receptor-like kinase 1
ALP	alkaline phosphatase
AMP	adenosine monophosphate
ANKH	progressive ankylosis homolog protein
ApoE	apolipoprotein E
ATP	adenosine triphosphate
AXL	AXL receptor tyrosine kinase
BMP	bone morphogenetic protein
Ca	calcium
Ca_5_(PO_4_)_3_OH	calcium hydroxyapatite
CAD	coronary artery disease
CKD	chronic kidney disease
CNV	choroidal neovascularization
COPD	chronic obstructive pulmonary disease
CTC	connective tissue calcifying disorder
DES	(iso)desmosine
EC	endothelial cell
ENPP1	ectonucleotide pyrophosphatase-phosphodiesterase 1
ER	endoplasmic reticulum
GACI	generalized arterial calcification of infancy
GAS6	growth arrest specific-6
GGCX	gamma-glutamyl carboxylase
Gla	γ-carboxylated glutamate
Glu	glutamate
GRP	gla-rich protein
HE	hematoxylin-eosin
IHC	immunohistochemistry
KS	Keutel syndrome
LRP1	lipoprotein receptor-related protein 1
MGP	matrix gla protein
MRP6	multi-drug resistance-associated protein 6
OC	osteocalcin
OPN	osteopontin
OSX	osterix
PAD	peripheral arterial disease
Pi	inorganic phosphate
PIVKA-II	protein induced by vitamin K absence or antagonist-II
PO_4_	phosphate
PPi	inorganic pyrophosphate
PXE	pseudoxanthoma elasticum
RUNX2	runt-related transcription factor 2
SMAD	small body size mothers against decapentaplegic
SNP	single nucleotide polymorphism
TGF-β	transforming growth factor-β
TLR	toll-like receptor
VC	vascular calcification
VEGF	vascular endothelial growth factor
Vit K	vitamin K
VKCFD	vitamin K-dependent coagulation factor deficiency
VKDP	vitamin K-dependent protein
VKORC1	vitamin K 2,3-epoxide reductase complex subunit 1
VSMC	vascular smooth muscle cell
WT	wild type
